# Chemometric Differentiation of Organic Honeys from Southeastern Türkiye Based on Free Amino Acid and Phenolic Profiles

**DOI:** 10.3390/foods14173105

**Published:** 2025-09-05

**Authors:** Semra Gürbüz, Şeyda Kıvrak

**Affiliations:** 1 Gastronomy and Culinary Arts, Faculty of Tourism, Mardin Artuklu University, Mardin 47080, Türkiye; 2 Department of Nutrition and Dietetics, Faculty of Health Sciences, Muğla Sıtkı Koçman University, Kötekli 48000, Türkiye

**Keywords:** organic honey, free amino acids, phenolic compounds, honey authenticity, geographical origin, chemometrics

## Abstract

Verifying the geographical origin of honey is crucial for its market value and for preventing fraudulent practices. This study aimed to characterize the chemical profiles of organic honeys from three distinct regions in Southeastern Türkiye—Şırnak Faraşin, Siirt Merkez, and Siirt Pervari—to establish a robust method for geographical authentication. A total of 51 multifloral honey samples were analyzed. The concentrations of 20 free amino acids (FAAs) and 16 phenolic compounds were quantified using (UPLC-ESI-MS/MS). The resulting data were subjected to both an unsupervised (PCA, CA) and supervised (PLS-DA, RF, SVM) chemometric analysis to identify biochemical markers for each region. The results revealed a distinct chemical fingerprint for each region. Based on the FAA profiles, the PLS-DA method provided the best overall classification, achieving an excellent discrimination with a total accuracy of 94.1% in the Şırnak Faraşin honeys. For the phenolic compound profiles, the RF method achieved the highest correct classification rate for Şırnak Faraşin honeys at 88.2%. This study demonstrates that an integrated approach, combining FAA and phenolic profiles with supervised chemometric methods, provides a successful and reliable model for determining the geographical origin of these multifloral honeys.

## 1. Introduction

Honey, a natural product prized for its flavor and nutritional value, offers a range of health benefits, including antioxidant, antimicrobial, and anti-inflammatory effects [1,2]. Its complex composition, encompassing carbohydrates, water, phenolic compounds, free amino acids, proteins, minerals, vitamins, enzymes, pigments, and sterols [3,4], is highly variable, influenced by factors such as the floral sources, regional geography and climate, production season, storage, and processing methods [5,6]. This variability, while contributing to the diversity of honey types, also creates opportunities for fraudulent practices, including the mislabeling of the botanical or geographical origin, adulteration with cheaper syrups or lower-quality honeys [7,8], and falsely declaring conventional products as organic [9]. With significant global trade [10], honey’s market value is often linked to its floral or geographic origin, organic and/or local production status [11,12], and registration with protected designations of origin (PDO) or protected geographical indications (PGIs) [13,14]. These economic factors underscore the critical need for robust authentication strategies. Honey’s diverse chemical profile, including free amino acids, volatile compounds, phenolic compounds, minerals, oligosaccharides, and proteins, provides a rich source of potential markers for determining the botanical and geographical origin [5,13,15,16,17,18,19,20].

Free amino acids (FAAs), linked to pollen—a key nutritional component for honeybees—have emerged as promising indicators of honey origin [15,21]. Amino acids are fundamental organic compounds involved in vital life processes such as biosynthesis and neurotransmission; they also serve as structural elements and energy sources essential for cell growth, differentiation, and function [22]. These FAAs, present in honey, originate from nectar, pollen, and honeybee secretions [23].

Phenolic compounds including phenolic acids and flavonoids are another key group of constituents that contribute significantly to honey’s antioxidant and therapeutic effects. Numerous studies have demonstrated that the phenolic profile and antioxidant capacity of honey vary according to its geographical and floral sources [16,17,18,19,24,25,26,27,28]. These components are credited with a wide range of health-protective activities, including anti-inflammatory, antimicrobial, anticancer, and antidiabetic effects [29,30,31].

To analyze such complex data, researchers employ chemometrics, defined as the use of mathematical and statistical techniques to extract important chemical data from complex data sets in studies on food adulteration [32].

Consequently, studies employing FAA and phenolic profiling, often combined with advanced analytical techniques like ultra-performance liquid chromatography with tandem mass spectrometry (UPLC-ESI-MS/MS) [33,34,35,36], have successfully differentiated honeys from various geographical locations [18,21,23,37,38,39,40]. These studies use a range of chemometric techniques, including unsupervised pattern recognition methods like Principal Component Analysis (PCA) and Cluster Analysis (CA), as well as supervised classification methods such as the Partial Least Squares Discriminant Analysis (PLS-DA), Random Forests (RFs), and Support Vector Machines (SVMs). Because the floral composition is influenced by environmental factors like the soil and climate [3,41], the FAA and phenolic profile can serve as an indicator of a honey’s specific production area. While some research has focused on origin distinction in monofloral and honeydew honeys [17,42,43,44,45], studies on multifloral honeys, particularly regarding the geographical origin, remain limited [18,21].

This is particularly true for Türkiye, the world’s second largest honey producer, which is known for its rich biological diversity and wide array of honeys [46,47]. Although a few studies have examined the properties of Turkish monofloral and honeydew honeys [35,36,48,49,50], no study has yet integrated both unsupervised and supervised chemometric techniques to differentiate multifloral honeys from specific Turkish regions, measure the performance of different classification methods, and determine the differential biochemical markers. Therefore, this study aims to fill this gap by investigating the FAA and phenolic compound profiles of organic honey samples from three distinct regions in Southeastern Anatolia, Türkiye: Şırnak Faraşin, Siirt Merkez, and Siirt Pervari. These regions were selected because of their historical, economic, and legal importance in Turkish honey production. Beekeeping has been practiced in these areas for centuries; notably, Siirt Pervari honey was consumed in the palace kitchens during the Ottoman Empire [51]. Both provinces were highlighted for organic beekeeping in the Southeastern Anatolia Region Organic Agriculture Cluster Project, which was launched in 2011 by the Ministry of Development with technical support from the United Nations Development Programme [52], and beekeeping continues to be a sustainable source of income for the rural population [53]. According to 2024 data from the Ministry of Agriculture and Forestry, Siirt province is ranked as the fifth largest honey producer in Türkiye, with 1379 enterprises and 3048 tons of honey production [54]. Siirt Pervari honey has been owned by the PDO since 2004 [55]. Şırnak province is also a significant producer, with 924 enterprises and 684 tons of production [54]. Honey from this sampling region was granted PGI status in May 2025 [56]. This study applies an integrated chemometric model, beginning with a statistical analysis followed by both unsupervised and supervised methods, with two primary objectives: first, to identify the key biochemical markers that enable the geographical differentiation of each region, and second, to compare the performance of three chemometrics methods (PLS-DA, RF, SVM) in order to establish a robust authentication model.

## 2. Materials and Methods

### 2.1. Study Area

**Figure 1:**
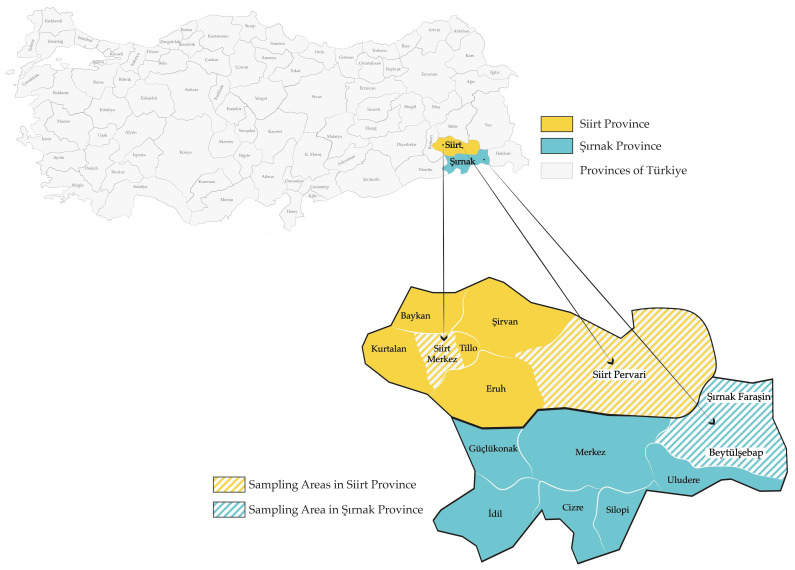
Geographical locations of honey sample collection areas.

This study was conducted in three distinct geographical regions in Southeastern Anatolia, Türkiye: Şırnak Faraşin, Siirt Merkez, and Siirt Pervari (Figure 1). These three sampling locations possess unique environmental characteristics.

Şırnak Faraşin is a high-altitude plateau (2625 m) in the Beytüşşebap district of Şırnak province (37.693295° N, 43.419288° E) characterized by diverse flora dominated by Apiaceae, Caryophyllaceae, Fabaceae, and Rosaceae [57]. In contrast, Siirt Merkez is located at a lower altitude (887 m) in the Merkez district of Siirt province (37.920280° N, 41.914720° E) that includes wild and cultivated species such as Asteraceae, Fabaceae, and Lamiaceae [58].

Siirt Pervari is situated at an intermediate altitude (1380) (37.932504° N, 42.547991° E) and has a floral composition similar to Siirt Merkez.

### 2.2. Honey Samples

A total of 51 organic, multifloral honey samples were collected directly from beekeepers in these three regions during the November–December harvest season with the support of the Şırnak Beekeepers Association and the Siirt Beekeepers Association. Samples (200–300 g each) were collected in sterilized glass jars and labeled with the collection date, region, and beekeeper information. The samples were transported to the laboratory and stored in the dark at 4 °C until analysis. To minimize variability due to storage conditions, all analyses were performed within three months of collection.

### 2.3. Analysis of Free Amino Acid

#### 2.3.1. Reagents and Chemicals for FAA Analysis

Analytical standards of 20 amino acids—alanine (Ala), asparagine (Asn), aspartic acid (Asp), arginine (Arg), cysteine (Cys), glycine (Gly), glutamine (Gln), glutamic acid (Glu), histidine (His), isoleucine (Ile), leucine (Leu), lysine (Lys), methionine (Met), phenylalanine (Phe), proline (Pro), serine (Ser), threonine (Thr), tyrosine (Tyr), tryptophan (Trp), and valine (Val)—were obtained from Sigma–Aldrich Chemie GmbH (Steinheim, Germany). Individual stock standard solutions of each amino acid were prepared in methanol at a concentration of 1000 mg/L. Methanol (LC/MS grade) and formic acid (≥99% purity), and all other solvents and reagents were of analytical grade and supplied by Merck (Darmstadt, Germany). Ultrapure water, generated using a Milli-Q water purification system (Advantage A10, Millipore, Molsheim, France) incorporating reverse osmosis, ion exchange, and final-stage filtration, was used for all solution preparations. Calibration standards ranging from 0 to 250 ng/mL were prepared by diluting the stock solutions with 20% methanol (*v*/*v*).

#### 2.3.2. Sample Preparation for FAA Analysis

Before analysis, honey samples were equilibrated to room temperature (25 °C). For each sample, 2.0 g of honey was accurately weighed into a 50 mL polypropylene centrifuge tube. Then 20 mL of a pre-prepared solution of 20% methanol (*v*/*v*) in ultrapure water, acidified with 0.1% formic acid (*v*/*v*), was added. The mixture was vortexed at maximum speed for 1 min to ensure complete dissolution and then sonicated in an ultrasonic bath at 36 °C for 10 min to facilitate extraction of the free amino acids. This temperature was chosen to optimize extraction efficiency while minimizing potential degradation. Following sonication, the samples were centrifuged at 4000× *g* rpm and 4 °C for 15 min to remove any particulate matter. The supernatant was carefully collected and filtered through a 0.20 μm pore diameter polytetrafluoroethylene (PTFE) syringe filter to remove any remaining solid particles. The filtered extracts were transferred into 2 mL HPLC vials and stored at −20 °C until analysis to prevent amino acid degradation.

#### 2.3.3. Free Amino Acid Analysis by UPLC-ESI-MS/MS

Free amino acid analysis was performed using an Acquity Ultra Performance Liquid Chromatography system (Waters Corporation, Milford, MA, USA) coupled with a Xevo TQ-S triple quadrupole mass spectrometer equipped with an electrospray ionization (ESI) source. Chromatographic separation was achieved on an Acquity UPLC BEH C18 column (1.7 µm, 2.1 × 100 mm; Waters Corporation) maintained at 40 °C. The injection volume was 1 µL. Mobile phase A consisted of 0.5% aqueous formic acid, while mobile phase B was methanol/water (50:50, *v*/*v*) containing 0.5% formic acid. A gradient elution program was employed at a flow rate of 0.4 mL/min

Mass spectrometry was performed in positive ESI mode using multiple reaction monitoring (MRM). The specific MRM transitions, cone voltages, and collision energies for each amino acid were optimized for maximum sensitivity and selectivity (see Supplementary Material S1, Tables S1 and S2). External calibration curves were constructed using standard solutions of each amino acid at concentrations ranging from 0 to 250 ng/mL. The limit of detection (LOD) and limit of quantification (LOQ) for each amino acid were determined based on signal-to-noise ratios of 3:1 and 10:1, respectively.

Importantly, the analytical method employed required no derivatization or additional cleanup steps, thereby enabling rapid and direct quantification of all 20 FAAs in complex honey matrices. This method was adapted from the validated protocol by [33,59], who reported the successful application of underivatized amino acid analysis using UPLC-ESI-MS/MS. The instrumental configuration included a column heater/cooler, binary solvent manager, and sample manager, integrated with the Xevo TQ-S system. All analytical parameters (including MS conditions and MRM transitions) were consistent with those reported by [33,59] and were applied without modification for the current analysis.

### 2.4. Analysis of Phenolic Compounds

The identification and quantification of phenolic compounds (phenolic acids and flavonoids) were performed using a validated UPLC-ESI-MS/MS method adapted from Kıvrak and Kıvrak [36]. The detailed analytical parameters for this method, including limits of detection (LoD), recovery rates, regression equations, and correlation coefficients, are provided in Supplementary Material S1, Table S3.

#### 2.4.1. Standards, Reagents, and Calibration

Analytical standards, including pyrogallol, homogentisic acid, 3,4-dihydroxybenzoic acid (protocatechuic acid), gallic acid, gentisic acid, pyrocatechol, galantamine, 4-hydroxybenzoic acid, 3,4-dihydroxybenzaldehyde, catechin hydrate, vanillic acid, caffeic acid, syringic acid, vanillin, epicatechin, catechin gallate, p-coumaric acid, ferulic acid, rutin, trans-2-hydroxycinnamic acid, myricetin, resveratrol, trans-cinnamic acid, luteolin, quercetin, naringenin, genistein, apigenin, kaempferol, hesperetin, chlorogenic acid, and chrysin, were purchased from Sigma-Aldrich Chemie GmbH (Steinheim, Germany). Stock solutions of each standard (1000 mg/L) were prepared in methanol. Mixed working standard solutions (50 mg/L) were then serially diluted to prepare calibration standards ranging from 0.00 to 10.00 mg/L.

#### 2.4.2. Sample Preparation and Extraction

For each of the 51 honey samples, 10 g was dissolved in 50 mL of ultrapure water and vortexed for 5 min. Liquid–liquid extraction was performed by adding 50 mL of ethyl acetate and shaking the mixture for 30 min (Promax 2020, Heidolph, Germany). After phase separation (180 min), the aqueous layer was re-extracted two additional times with ethyl acetate. The pooled organic extracts were evaporated to dryness under vacuum at 36 °C using a rotary evaporator (Heidolph, Germany). The final residue was reconstituted in 5 mL of methanol and filtered through a 0.20 µm PTFE syringe filter (Chromafil Xtra, Macherey-Nagel, Düren, Germany) prior to analysis.

#### 2.4.3. UPLC-ESI-MS/MS Instrumentation and Conditions

Analyses were conducted on a Waters Acquity UPLC system coupled with a Xevo TQ-S triple quadrupole mass spectrometer with an electrospray ionization (ESI) source (Waters, Milford, MA, USA). Chromatographic separation was performed on a Waters BEH C18 column (2.1 × 100 mm, 1.7 µm) maintained at 40 °C. The mobile phases consisted of 0.5% (*v*/*v*) acetic acid in water (A) and 0.5% (*v*/*v*) acetic acid in acetonitrile (B). A 2 µL aliquot was injected and eluted using a linear gradient at a flow rate of 0.65 mL/min: 0–1 min, 99%. A for 1 min, ramped to 70% A over 9 min, ramped to 5% A over 2 min, and finally returned to 99% A for 1 min to re-equilibrate the column.

### 2.5. Data Analysis

Raw UPLC-ESI-MS/MS data were processed using MassLynx software (MassLynx V 4.1 SCN 810, Waters Corporation), and the concentration of each FAA and phenolic compound was calculated from its respective calibration curve.

All subsequent statistical analyses were performed using R software (version 4.0.2; R Foundation for Statistical Computing, Vienna, Austria). A *p*-value of <0.05 was considered statistically significant. Due to the non-normal distribution of the data, non-parametric tests were employed. To compare compound concentrations among the three geographical regions, the Kruskal–Wallis test was used, with results presented as median and interquartile range (Q). Significant differences were further investigated using the Conover post hoc test with a Bonferroni correction for pairwise comparisons.

To explore patterns and relationships between chemical profiles and geographical origins, several chemometric techniques were applied. Prior to multivariate analysis, the logarithmic transformation of all variables was performed, and the data matrix was pre-processed using autoscaling (mean-centering and scaling to unit variance) to ensure that all variables contributed equally to the model, regardless of their absolute concentration. For initial exploratory analysis, unsupervised methods were used. Principal Component Analysis was used to reduce dimensionality and visualize sample clustering. Hierarchical Cluster Analysis (CA) was performed using Ward’s method with Euclidean distance to group samples based on chemical similarity. Additionally, heatmaps were generated using the heatmap package in R to visualize concentration variations in individual compounds across all samples.

Finally, to build a robust authentication model, three supervised classification methods were implemented. First, all variables were converted to numerical format and standardized to have a mean of zero and a variance of one. Class labels were composed of three regions (Şırnak Faraşin, Siirt Merkez, Siirt Pervari).

PLS-DA analysis was performed using the mixOmics package in R. The number of latent variables (LVs) was scanned between 1 and 5, and the selection process was performed using iterative M-fold cross-validation (5-fold, 10 replicates, seed = 123). Balanced Error Rate (BER) was used as the selection criterion, and the max.dist method was used as the classification rule.

In the Random Forest analysis, the model was built with the randomForest and caret packages, and 500 trees (ntree = 500) were used. The number of randomly selected variables (mtry) in each split was scanned between 2 and √p + 2, and the optimum value was determined using 5-fold cross-validation. In the Support Vector Machines (RBF kernel) analysis, hyperparameters were optimized using grid search. The C (cost) parameter was scanned between 2^−3^ and 2^7^, and the gamma parameter was scanned between 2^−7^ and 2^3^, and the optimal combination was selected after 5-fold cross-validation. In all models, set.seed (123) was used to reduce the effect of randomness.

Model performance was assessed through k-fold cross-validation, and the evaluation metrics included accuracy, precision, recall (sensitivity), F1-score, Matthews correlation coefficient (MCC), and the area under the ROC curve (AUC). Variable importance scores were also obtained to determine the most influential compounds contributing to the classification.

## 3. Results and Discussion

### 3.1. Determination of Free Amino Acid Content

**Figure 2:**
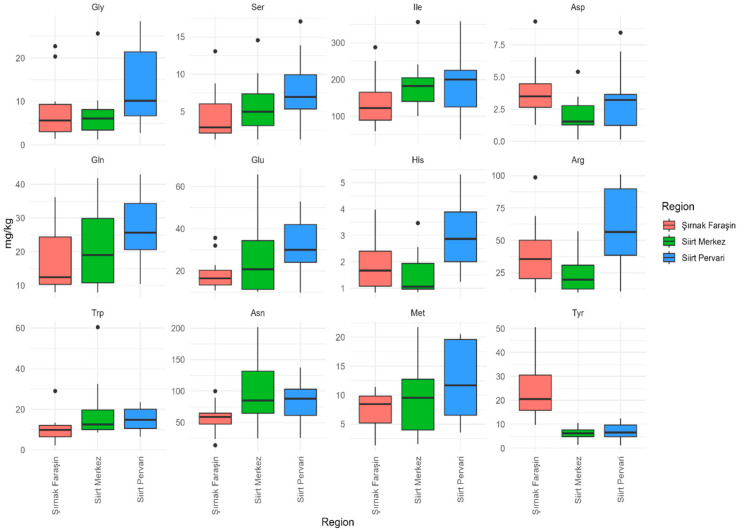
Box plots showing the regional distribution of FAAs with statistically significant concentration differences (*p* < 0.05) among the three regions. The line within the box indicates the median, the boxes indicate Q1 and Q3, the lines indicate the minimum and maximum values, and the dots indicate outliers.

Significant regional differences were observed in the FAA profiles of honey samples from Şırnak Faraşin, Siirt Merkez, and Siirt Pervari (Table 1). Pairwise comparisons based on the Conover post hoc test revealed that honey samples from Şırnak Faraşin significantly differed from those of Siirt Merkez in concentrations of Asn (*p* = 0.016), Asp (*p* = 0.014), Tyr (*p* < 0.001), and Trp (*p* = 0.013). On the other hand, significant differences were observed between Şırnak Faraşin and Siirt Pervari for Gly (*p* = 0.013), Ser (*p =* 0.036), Ile (*p* = 0.043), Asn (*p* = 0.016), Asp (*p =* 0.014), Gln (*p =* 0.024), Glu (*p* = 0.010), Met (*p* = 0.047), His (*p* < 0.001), Arg (*p* = 0.001), Tyr (*p* < 0.001), and Trp (*p* = 0.013). Comparatively fewer differences were observed between Siirt Merkez and Pervari, limited to Gly (*p* = 0.013), His (*p* < 0.001), and Arg (*p* = 0.001) (Table 1, Figure 2). These patterns align with previous reports of the FAA variation across geographical origins, such as floral honeys from Argentina [21] and honeydew honeys from Brazil [60], reinforcing the utility of FAA profiling as a marker of origin.

The median total FAA content was 1271.49 mg/kg in Şırnak Faraşin honeys and was higher in Siirt regions (1569.45 mg/kg in Siirt Merkez; 1560.03 mg/kg in Siirt Pervari). These values are comparable to Kazakh honeys (1449.6 mg/kg) and notably exceed those reported in Poland, Belarus, Estonia, and Slovakia [4,35,61]. Such elevated levels suggest that the diverse flora of Southeastern Türkiye provides particularly amino acid-rich nectars.

Proline (Pro) was the dominant FAA across all regions, contributing ~31–33% of the total pool, consistent with its origin in the bee metabolism [44]. Median Pro values (410.3–522.5 mg/kg) were lower than Chinese grassland honeys [62], similar to Turkish multifloral honeys [46], and higher than Belarusian and Slovak honeys [35], confirming high honey maturity [61].

Leucine (Leu) was the second most abundant FAA (201.2–300.5 mg/kg), with concentrations exceeding those reported in Estonian, Polish, Slovak, and French honeys [4,35,42] but comparable to Italian buckwheat honey [63].

The concentrations of several amino acids varied significantly between the regions (Figure 2). For example, Gly, Arg, and Met levels were found to be particularly high in the Siirt Pervari honey compared to the other regions. In contrast, while relatively low Trp values were observed in the Şırnak Faraşin honey, Tyr was found to be higher. These distinct differences are likely attributable to variations in floral sources, edaphoclimatic conditions, and apicultural practices, all of which influence the amino acid composition of honey.

Essential FAAs (Val, Thr, Leu, Ile, Lys, Met, His, Phe, and Trp) were abundant, with Siirt Merkez showing the highest total value (878.72 mg/kg), followed by Siirt Pervari (860.13 mg/kg) and Şırnak Faraşin (700.64 mg/kg). These values are greater than those in Chinese grassland honeys and multifloral honeys from Kazakhstan and Poland [61,62]. The ratio of essential FAAs was highest in the Şırnak Faraşin honey (59.81%). Branched-chain amino acids (Val, Leu, and Ile) accounted for over half of the essential pool, particularly in Siirt Merkez, suggesting regional differences in floral inputs.

Distinct markers were also observed. Siirt Pervari honeys were characterized by elevated Met, which was significantly higher than in Şırnak Faraşin, contrasting with the trace or absent Met in French, Estonian, and Polish honeys [4,35,42].

Cysteine was detected in low amounts (0.46–1.01 mg/kg) and absent in some samples, consistent with earlier studies [4,33]. Notably, Tyr and Asp were uniquely enriched in Şırnak Faraşin, providing a robust regional fingerprint. Tyr levels (20.5 mg/kg) exceeded those reported in Turkish honeydew honeys [50] and were lower than those reported in Spanish and Chinese monofloral honeys [44,62], while Asp values, though lower than Brazilian and Polish honeys [3,35], were consistently higher than in Siirt samples.

Overall, the FAA profiles highlight clear biochemical differentiation among the three regions. The consistently high levels of essential FAAs underscore the nutritional value of these honeys, while specific enrichments of Tyr and Asp in Şırnak Faraşin and Gly, Ile, and Met in Siirt Pervari demonstrate the potential of FAA profiling for geographical authentication.

### 3.2. Principal Component Analysis and Cluster Analysis for Free Amino Acids

The Principal Component Analysis is an unsupervised multivariate statistical technique used to reduce data dimensionality while preserving most of the variance, making it useful for visualizing natural groupings and identifying discriminating variables [64].

Following the concentration and statistical analyses, chemometric methods such as the PCA and CA were employed to further investigate the variations in the amino acid profiles of the honey samples [3,21,39,43,60].

**Figure 3:**
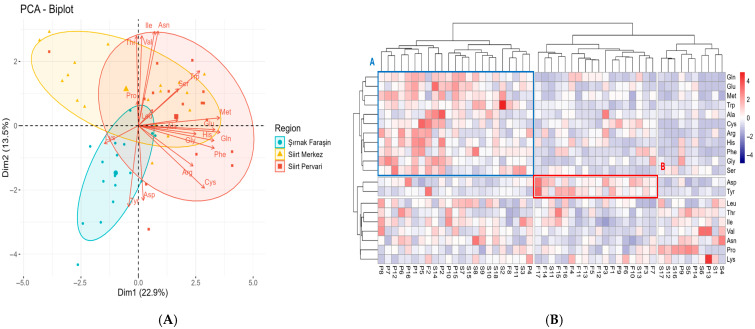
Principal Component Analysis biplot and heatmap visualization of FAA profiles in honey samples from three regions. (**A**) PCA biplot of FAA profiles. (**B**) Heatmap visualization of FAA concentrations. (F) Şırnak Faraşin, (S) Siirt Merkez, and (P) Siirt Pervari. Color intensity reflects relative abundance, with red indicating higher and blue indicating lower concentrations. (A) shows the location where Siirt honey is clustered, (B) shows the location where Şırnak Faraşin honey is clustered.

The analysis revealed that honey samples from the Şırnak Faraşin region were distinctly separated from those of Siirt Merkez and Siirt Pervari, indicating marked regional differences in the FAA profiles. This separation was driven by the higher concentrations of Tyr and Asp in the Şırnak Faraşin honey, which were identified as characteristic features of this regional honey profile (Figure 3A).

While the first two principal components shown in the visual biplot (Figure 3A) explained 36.4% of the cumulative variance, the full analysis was more robust. The complete model identified seven components with eigenvalues greater than one that collectively explained 68.28% of the total variance, confirming that the FAA profile is a strong basis for differentiation (see Supplementary Material S2, Table S1).

To further explore the clustering patterns of honey samples based on the amino acid composition, a Cluster Analysis was performed and visualized using a heatmap (Figure 3B). The heatmap reveals distinct amino acid profiles for each region. Honeys from Siirt Merkez and Siirt Pervari generally exhibit higher concentrations of certain FAAs, such as Gln, Glu, and Gly, while Şırnak Faraşin honeys are distinguished by relatively lower levels of these FAAs.

The results indicate that the distinguishing feature of Şırnak Faraşin honeys is their higher levels of Tyr and Asp, which supports the findings from the PCA. In contrast, honeys from the Siirt Merkez and Siirt Pervari regions displayed a heterogeneous structure, without a clear separation between the two. While these honeys consistently showed lower levels of Asp and Tyr, they exhibited variability in the concentration of other amino acids, with some samples being rich in certain amino acids, while others were comparatively lower (Figure 3A). These findings highlight the complex nature of the amino acid composition in the honeys from these two regions.

The clustering pattern highlights the distinct amino acid profiles of Şırnak Faraşin honeys, particularly with respect to Tyr and Asp, supporting their potential role as regional markers.

### 3.3. Identifying Regional Fingerprints for FAA Using Supervised Classification Methods

To move beyond the exploratory analysis of the PCA and build a robust classification model, three supervised chemometric methods—PLS-DA, RFs, and SVMs—were employed to validate the discrimination power of the FAA profiles. A comparison of the classification performance for each method is presented in Table 2, while detailed data on the most important FAA markers identified by each method are available in Supplementary Material S3, Tables S1, S3 and S5.

To build a robust classification model, the performances of the PLS-DA, RF, and SVM methods were compared using k-fold cross-validation. A comparison of the models showed that the PLS-DA method provided the highest overall classification success, achieving the best total accuracy (82.4%), F1-score (81.5%), and MCC of 0.735. The Random Forest method ranked second with a total accuracy of 76.5%, while the SVM method had the lowest overall performance with a total accuracy of 70.6%. All models were highly effective at identifying the honey from Şırnak Faraşin. The PLS-DA model, in particular, provided excellent discrimination for this group, with 94.1% accuracy and a perfect recall of 1.000.

An analysis of the variable importance was conducted for each method to identify the key biochemical markers responsible for separating the regions. Tyrosine was unequivocally the most powerful and consistent marker across all three methods. It had the highest loading values in the PLS-DA analysis and achieved importance scores of nearly 100% in both the RF and SVM methods for distinguishing the Şırnak Faraşin honey. Other amino acids, including His, Arg, Glu, and Gly, also contributed significantly to the models’ discrimination power (the detailed analysis data are provided in Supplementary Material S3, Tables S1, S3 and S5).

This study demonstrates that the FAA profiles of multifloral honeys from Southeastern Türkiye vary significantly by region and can be used for geographical classification with a high success rate. The methods’ ability to classify the Şırnak Faraşin honey with a higher accuracy than the two Siirt honeys is likely explained by distinct geographical factors. The Şırnak Faraşin region is located in a different province, at a higher altitude, and is more geographically distant, likely resulting in a unique floral environment that produces a more distinct FAA fingerprint in its honey.

### 3.4. Determination of Phenolic Compound Profile

Of the 32 phenolic compounds targeted, 16 were consistently quantified, including 10 phenolic acids and 6 flavonoids (Table 3). Several phenolic acids—homogentisic, 3,4-dihydroxybenzoic (protocatechuic), gentisic, vanillic, caffeic, and p-coumaric—were detected in all samples, forming the core phenolic backbone of honeys from these regions of Türkiye.

**Figure 4:**
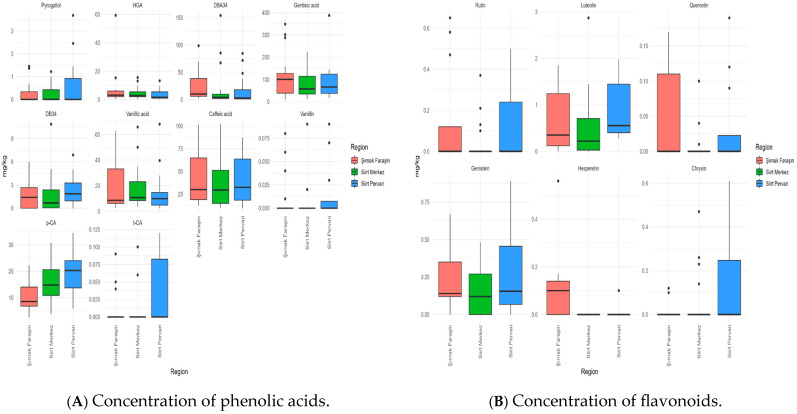
Box plots comparing the concentrations of individual phenolic acids (**A**) and flavonoids (**B**) in honeys. The line within the box indicates the median, the boxes indicate Q1 and Q3, the lines indicate the minimum and maximum values, and the dots indicate outliers.

Phenolic acids overwhelmingly dominated the profile, constituting > 98% of the total phenolic content (TPC). The TPC ranged from 121.6 mg/kg in Siirt Merkez to 164.7 mg/kg in Şırnak Faraşin, values that are substantially higher than those reported for many European and Asian honeys, including Spanish multifloral (11–99 µg/g) [16] and Chinese floral honeys (22.9–111.6 µg/g) [19]. Gentisic acid was the most abundant compound (Figure 4A), contributing nearly half of the total phenolics (Supplementary Material S4, Table S1). and reaching 100.6 mg/kg in Şırnak Faraşin. Together with caffeic acid (29.5–32.5 mg/kg), these compounds established the common biochemical fingerprint of the honeys studied.

Despite this shared foundation, statistical analyses revealed clear inter-regional markers. p-Coumaric acid was significantly higher in Siirt honeys than in Şırnak Faraşin (*p* = 0.003). Protocatechuic acid was elevated in Şırnak Faraşin compared to Siirt Pervari (*p* = 0.049). Vanillic acid reached its highest levels in Siirt Merkez, aligning with values for specific Turkish floral honeys, such as sideris and lavender [36]. These differences demonstrate that even within a multifloral context, phenolic acids can provide robust markers of origin [17,50].

Although minor in concentration, flavonoids contributed additional discriminatory power. The total flavonoid content was highest in Siirt Pervari (1.52 mg/kg), followed by Şırnak Faraşin (1.46 mg/kg), and was lowest in Siirt Merkez (0.82 mg/kg). Luteolin was a key marker in Siirt Pervari (*p* = 0.034), while rutin characterized Şırnak Faraşin, accounting for nearly 40% of its flavonoid fraction (Supplementary Material S4, Table S2). These findings are notable, since rutin is often absent in other Turkish honeys, such as acacia and chestnut [36], yet is a potent antioxidant with anticancer potential [65]. An additional qualitative variation included the absence of hesperetin in Siirt Merkez (but presence in the other regions, consistent with Malaysian honeys [66]) and differing contributions of chrysin, quercetin, and genistein.

In summary, the phenolic profiles of these honeys are defined by a stable backbone of gentisic and caffeic acids but are differentiated by significant variations in p-coumaric, protocatechuic, and vanillic acids, as well as by region-specific flavonoids such as luteolin and rutin (Figure 4A,B). These distinct signatures confirm the utility of phenolic profiling as a powerful tool for both the geographical authentication and nutritional characterization of honeys from Southeastern Türkiye.

### 3.5. Principle Component Analysis and Cluster Analysis of Phenolic Profiles

To visualize the complex relationships between the 16 quantified phenolic compounds and the geographical origin of the honeys, the PCA and heatmap visualization were performed separately for the phenolic acid and flavonoid profiles, as well as for the combined data set, to identify the key drivers of regional differentiation.

#### 3.5.1. Differentiation Based on Phenolic Acids

**Figure 5:**
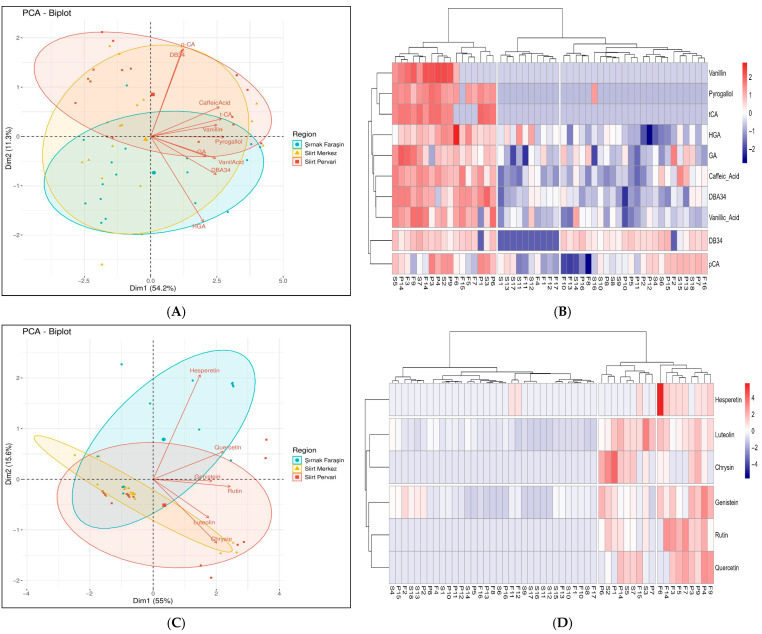

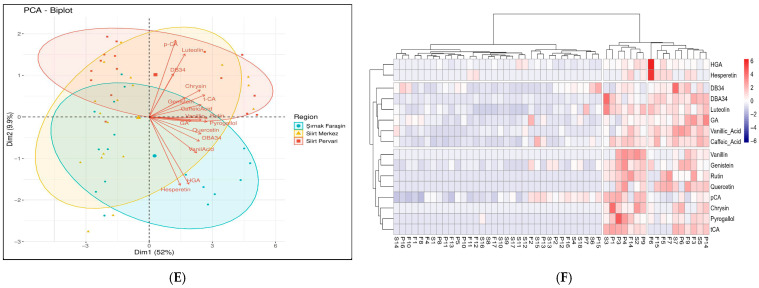
(**A**,**C**,**E**) shows PCA biplots and (**B**,**D**,**F**) shows heatmap visualization for FAAs’ phenolic acids, flavonoids, and combined phenolic compounds. In the heatmap visualization geographical origins are labeled as follows: (F) Şırnak Faraşin, (S) Siirt Merkez, and (P) Siirt Pervari. (**A**) PCA biplot of the phenolic acid profiles. (**B**) Heatmap of phenolic acid concentration, for individual honey sample. (**C**) PCA biplot of flavonoid profiles. (**D**) Heatmap of flavonoid concentration, for individual honey sample. (**E**) PCA biplot of the combined phenolic acid and flavonoid profiles. (**F**) Heatmap of the combined phenolic compounds.

The Principal Component Analysis of the 10 phenolic acids revealed that the first three components explained 76.2% of the total variance, confirming the profile’s utility for regional discrimination (Supplementary Material S2, Table S2). The PCA biplot (Figure 5A) showed that the Siirt Merkez and Siirt Pervari samples formed a relatively cohesive cluster, whereas the Şırnak Faraşin samples were more widely dispersed, suggesting greater chemical heterogeneity. This separation was primarily driven by the higher concentrations of p-coumaric and caffeic acids in the Siirt honeys. This was visually confirmed by the heatmap analysis (Figure 5B), which showed consistently higher levels of p-coumaric and caffeic acids in the Siirt samples. The heatmap also highlighted the greater chemical diversity within the Şırnak Faraşin group, particularly the high variability in the concentrations of gentisic acid, 3,4-dihydroxybenzoic acid, and vanillic acid, which explains their more scattered distribution in the PCA plot. The biological significance of this diverse phenolic content is substantial, as it suggests a richer profile with a potentially enhanced antioxidant capacity and other health-protective functional properties, such as potential anti-inflammatory or anticancer effects.

#### 3.5.2. Differentiation Based on Flavonoids

The flavonoid profile provided an even more powerful tool for discrimination. The Principle Component Analysis of the six quantified flavonoids explained a remarkable 82.1% of the total variance in its first three components (Supplementary Material S2, Table S3). The PCA biplot (Figure 5C) showed a distinct and unambiguous separation of the Şırnak Faraşin samples from the two Siirt regions. The heatmap (Figure 5D) visually reinforced this finding, displaying a clear block of high rutin and hesperetin levels almost exclusively in the Şırnak samples. This is particularly significant, as the high abundance of rutin, a flavonoid known for its potent antioxidant and cardiovascular-protective effects [65,67], enhances the potential functional value of the Şırnak Faraşin honey.

#### 3.5.3. The Integrated Analysis of the Complete Phenolic Profile

When all 16 phenolic compounds were analyzed together, the PCA explained 70.2% of the total variance in the first three components, providing a holistic view of the chemical differences (Supplementary Material S2, Table S4). The integrated PCA biplot (Figure 5E) and heatmap (Figure 5F) confirmed the overall trend: the Siirt Merkez and Siirt Pervari honeys share a similar chemical profile and cluster together, while the Şırnak Faraşin honeys form a distinct, more heterogeneous group. This comprehensive analysis demonstrates that while these regions share a common phenolic backbone—evidenced by the consistent presence of compounds like caffeic acid, vanillic acid, and genistein—it is the unique combination of differentiating markers that allows for successful geographical authentication. The use of multiple chemical markers for discrimination is a robust strategy that has proven effective in other honey origin studies [17]. The clear patterns observed in the PCA and heatmap analyses confirm that the full phenolic profile is a powerful tool for verifying the geographical origin of these high-value Turkish honeys.

### 3.6. Identifying Regional Fingerprints for Phenolic Compounds Using Supervised Classification Methods

Based on initial exploratory analyses (Kruskal–Wallis, PCA, and CA), which indicated that phenolic compounds could provide information about the geographical origin, supervised classification methods (PLS-DA, RF, and SVM) were performed to create a model for geographic classification by determining biochemical markers.

The results of the classification analyses based on phenolic compounds are presented in Table 4. In terms of the overall accuracy, the RF method provided the highest success rate with 58.8%, while the PLS-DA and SVM showed a similar performance at 54.9%. When examined by region, the models showed varying strengths. For Şırnak Faraşin honeys, the RF method achieved the highest correct classification rate at 88.2%, followed by the PLS-DA (82.4%) and SVM (80.4%). For Siirt Merkez honeys, the RF and SVM performed equally with a 64.7% accuracy, while for the Siirt Pervari honey, the PLS-DA achieved the highest classification success at 70.6%. A comparison of sensitivity and F1 scores showed that the RF had a more balanced performance for Şırnak Faraşin and Siirt Pervari honeys. The PLS-DA and RF showed similar error levels, while the SVM exhibited higher error rates in some groups, especially Siirt Pervari.

When the PLS-DA component loadings of the phenolic acid components are examined, the first component (comp1) explains 13% of the total variance, while the second component (comp2) explains 44%. The compounds with the highest loading values in the first component are p-CA (2.208), hesperetin (2.027), and chrysin (1.424), which stand out as the elements with the strongest discriminatory properties in the first component of the method. Other compounds with high loading values are t-CA (1.091), gentisic acid (0.910), and HGA (0.857). In the second component, p-CA (2.047) and hesperetin (1.936) again have the highest loading values, demonstrating a common and strong discriminatory property in both components.

When the contribution levels of phenolic compounds to the classification success were examined in the RF analysis, hesperetin was determined as the compound with the highest importance across all regions (overall importance: 76.54%). Luteolin (60.31% overall importance) and DBA34 (59.65%) were other high-importance compounds. When evaluated regionally, hesperetin (100%), p-CA (74.44%), and DBA34 (73.52%) were prominent in Şırnak Faraşin samples. In Siirt Merkez samples, hesperetin (87.64%), luteolin (64.13%), and gentisic acid (51.29%) were the most prominent compounds. For Siirt Pervari samples, luteolin (78.12%), DBA34 (67.99%), and HGA (54.30%) were notable for their high significance levels.

According to the variable importance analysis conducted with the SVM, p-CA stood out as the compound with the highest average importance (89.94%) across all regions. It had the highest value of 100% in the Şırnak Faraşin and Siirt Pervari groups, while it was determined as 69.82% in Siirt Merkez. Hesperetin (76.54% overall importance) and luteolin (66.21%) were identified as other highly important compounds. On a regional basis, p-CA (100%), hesperetin (80.25%), and DBA34 (62.96%) stood out in the Şırnak Faraşin samples. In Siirt Merkez, hesperetin (80.25%), luteolin (76.47%), and p-CA (69.82%) were prominent, while in the Siirt Pervari samples, p-CA (100%), luteolin (76.47%), and DBA34 (62.96%) were the most prominent compounds.

The analyses yielded biochemical markers for each region that can be used to distinguish the honeys. The phenolic compound profiles in the examined multifloral honeys differed by region, and this difference could be classified according to the geographical origin using multivariate statistical analyses. The correct classification rates for honeys from the Şırnak Faraşin region ranged from 80.4% to 88.2%, which is higher than for honeys from the other regions (Supplementary Material S3, Tables S2 and S6).

## 4. Conclusions

This study successfully characterized the FAA and phenolic compound profiles of organic multifloral honeys from three distinct regions in Southeastern Türkiye. The results confirmed that these honeys possess rich and varied chemical profiles, attributable to their high concentrations of FAAs and phenolic compounds, which reflects the unique local bee flora and environmental conditions.

The chemometric analysis revealed a distinct chemical fingerprint for each region. Supervised classification methods successfully authenticated the honeys; the PLS-DA was most effective (94.1%) for the FAA profiles, while the RF showed the highest accuracy (88.2%) for identifying the Şırnak Faraşin honey based on its phenolic profile. The high classification accuracy for the Şırnak Faraşin honey is likely due to its unique high-altitude microclimate, whereas the greater chemical similarity between the two Siirt honeys reflects their geographical proximity.

This research validates that the integrated analysis of FAA and phenolic compound profiles, interpreted through robust chemometric methods, provides a reliable and effective methodology for verifying the geographical origin of these honeys. This approach offers a strong scientific basis for protecting regional value, ensuring product authenticity, and combating fraudulent practices in the honey industry.

Finally, the model presented here is successful and promising for the authentication of multifloral honeys from the Southeastern Anatolia region. It is expected that these results will contribute to future work on preventing adulteration related to honey’s origin. Further testing of this integrated model on honeys from different floral and geographical origins is recommended to support its validation and broader application

## Figures and Tables

**Table 1:** Comparison of free amino acid concentrations in honey samples from three geographical regions. Values are presented as median [Q1: First quarter, Q3: Third quarter] in mg/kg.

Free Amino Acids (FAAs)	Şırnak Faraşin (*n* = 17)	Siirt Merkez (*n* = 18)	Siirt Pervari (*n* = 16)	*p*-Value *
Gly	5.63 [2.76, 9.33] ^a^	6.09 [3.28, 8.41] ^a^	10.16 [6.61, 22.11] ^b^	**0.013**
Ala	3.56 [2.28, 6.33]	4.18 [2.44, 10.29]	6.37 [3.85, 9.51]	0.110
Ser	2.87 [2.08, 6.27] ^a^	4.96 [2.83, 8.01] ^ab^	6.95 [4.35, 11.35] ^b^	**0.036**
Pro	410.3 [310.9, 552,3]	522.5 [333.5, 759.6]	495.5 [365.9, 628.7]	0.223
Val	15.94 [14.12, 30.45] ^a^	26.96 [17.43, 46.92] ^ab^	31.47 [15.34, 43.43] ^b^	0.059
Thr	12.33 [9.6, 14.41]	14.4 [10.6, 20.3]	14.79 [8.9, 22.8]	0.320
Cys	0.99 [0.62, 1.89]	0.46 [0, 1.56]	1.01 [0.71, 1.51]	0.156
Leu	201.2 [146.7, 235.9]	300.5 [138.1, 378.8]	242 [158, 317.2]	0.242
Ile	122.4 [78.8, 180.4] ^a^	182.3 [133.5, 212.5] ^ab^	199.8 [124.9, 244.9] ^b^	**0.043**
Asn	58.9 [42.5, 73.8] ^a^	85.1 [61.9, 133.8] ^b^	87.9 [46.4, 103.6] ^b^	**0.016**
Asp	3.5 [2.61, 4.64] ^a^	1.54 [1.17, 2.85] ^b^	3.21 [1.22, 3.77] ^ab^	**0.014**
Lys	199.9 [100.4, 215.1]	171.8 [119.6, 223.1]	142.3 [124.8, 195.8]	0.706
Gln	12.5 [10.2, 24.9] ^a^	19.1 [10.1, 31.1] ^ab^	25.7 [20.1, 36.5] ^b^	**0.024**
Glu	16.5 [13, 21.4] ^a^	20.8 [10.8, 35.7] ^ab^	30 [23.3, 42.6] ^b^	**0.010**
Met	8.4 [4.5, 9.9] ^a^	9.5 [3.7, 12.9] ^ab^	11.7 [6.1, 19.7] ^b^	**0.047**
His	1.67 [1.03, 2.67] ^a^	1.06 [0.96, 2.04] ^a^	2.87 [1.99, 3.97] ^b^	**<0.001**
Phe	128.9 [126.4, 225.7]	159.6 [100.5, 196.9]	200.3 [142.2, 247.3]	0.094
Arg	35.6 [20.3, 50.7] ^a^	19.7 [12.1, 34.8] ^a^	56.5 [33.4, 90.2] ^b^	**0.001**
Tyr	20.5 [14.7, 32.1] ^a^	6.3 [4.5, 8.1] ^b^	6.6 [3.9, 9.8] ^b^	**<0.001**
Trp	9.9 [6.4, 12.5] ^a^	12.6 [10, 20.6] ^b^	14.9 [9.9, 20.4] ^b^	**0.013**
Total FAAs	1271.49	1569.45	1560.03	
Total Essential FAAs	700.64	878.72	860.13	

* Superscript letters (a, b) within the same row indicate statistically significant differences among regions (*p* < 0.05), based on the Kruskal–Wallis test followed by Conover post hoc analysis.

**Table 2:** Performance metrics for supervised classification methods based on FAA profiles.

Methods	Class (Regions)	Predicted			Total	Accuracy	Precision (PPV)	Recall (TPR)	False Positive Rate (FPR)	False Discovery Rate (FDR)	F1-Score	Matthews Correlation Coefficient (MCC)	Negative Predictive Value (NPV)	True Negative Rate (TNR)	False Negative Rate (FNR)	AUC
		Şırnak Faraşin	Siirt Merkez	Siirt Pervari												
PLS-DA	Şırnak Faraşin	17	0	0	17	0.941	0.850	1.000	0.088	0.150	0.919	0.880	1.000	0.912	0.000	0.979
	Siirt Merkez	1	15	2	18	0.863	0.789	0.833	0.121	0.211	0.811	0.704	0.906	0.879	0.167	0.862
	Siirt Pervari	2	4	10	16	0.843	0.833	0.625	0.057	0.167	0.714	0.621	0.846	0.943	0.375	0.838
	Total	20	19	12	51	0.824	0.824	0.819	0.089	0.176	0.815	0.735	0.917	0.911	0.181	0.893
RF	Şırnak Faraşin	15	2	0	17	0.902	0.833	0.882	0.088	0.167	0.857	0.783	0.939	0.912	0.118	0.969
	Siirt Merkez	1	13	4	18	0.804	0.722	0.722	0.152	0.278	0.722	0.571	0.848	0.848	0.278	0.838
	Siirt Pervari	2	3	11	16	0.824	0.733	0.688	0.114	0.267	0.710	0.584	0.861	0.886	0.313	0.839
	Total	18	18	15	51	0.765	0.763	0.764	0.118	0.237	0.763	0.646	0.883	0.882	0.236	0.882
SVM	Şırnak Faraşin	13	1	3	17	0.902	0.929	0.765	0.029	0.071	0.839	0.777	0.892	0.971	0.235	0.931
	Siirt Merkez	1	10	7	18	0.765	0.714	0.556	0.121	0.286	0.625	0.465	0.784	0.879	0.444	0.759
	Siirt Pervari	0	3	13	16	0.745	0.565	0.813	0.286	0.435	0.667	0.491	0.893	0.714	0.188	0.820
	Total	14	14	23	51	0.706	0.736	0.711	0.145	0.264	0.710	0.578	0.856	0.855	0.289	0.837

**Table 3:** Concentrations of phenolic compounds (mg/kg) in honeys from three geographical regions.

Phenolic Acids	Şırnak Faraşin (mg/kg) (*n* = 17)	ND Count	Siirt Merkez (mg/kg) (*n* = 18)	ND Count	Siirt Pervari (mg/kg) (*n* = 16)	ND Count	*p*-Value *
Pyrogallol	0.69 [0.29, 1.38]	11	0.67 [0.58, 1.10]	13	1.23 [0.76, 2.76]	10	-
HGA	3.14 [1.71, 6.04]		2.81 [2.03, 6.37]		1.6 [1.03, 5.47]		0.408
DBA34	10.2 [5.76, 43.3] ^a^		4.04 [1.93, 12.1] ^ab^		2.71 [1.11, 31.7] ^b^		**0.049**
Gentisic acid	100.6 [38.6, 143.2]		57.5 [34.3, 120.1]		66.5 [37.7, 124.5]		0.301
DB34	1.4 [0, 3.05]	6	0.68 [0, 2.57]	5	1.86 [0.91, 3.67]	1	0.253
Vanillic acid	8.52 [5.67, 34]		10.7 [8.1, 27.1]		9.83 [4.63, 14.7]		0.506
Caffeic acid	30 [17.2, 66.9]		29.5 [14.4, 58.5]		32.5 [15.1, 63.8]		0.930
Vanillin	0.05 [0.02, 0.07]	13	0.02 [0.02, 0.09]	15	0.07 [0.04, 0.09]	12	-
p-CA	8.54 [6.45, 14] ^a^		14.78 [10.3, 21.1] ^b^		20.3 [12.6, 24.9] ^b^		**0.003**
t-CA	0.05 [0.04, 0.09]	14	0.08 [0.06, 0.10]	14	0.10 [0.07, 0.11]	10	-
**Total phenolic acids**	163.19		120.78		136.7		
**Flavonoids**							
Rutin	0.58 [0.30, 0.65]	12	0.17 [0.11, 0.33]	14	0.30 [0.20, 0.39]	10	-
Luteolin	0.35 [0.12, 1.3] ^ab^	2	0.22 [0, 0.79] ^a^	5	0.55 [0.38, 1.58] ^b^		**0.034**
Quercetin	0.14 [0.12, 0.16]	12	0.07 [0.02, 0.10]	14	0.12 [0.10, 0.17]	12	-
Genistein	0.14 [0.11, 0.4]	1	0.12 [0, 0.29]	7	0.15 [0.02, 0.52]	4	0.435
Hesperetin	0.14 [0.12, 0.16]	8	-	18	0.10 [0.10, 0.10]	14	-
Chrysin	0.11 [0.10, 0.12]	15	0.24 [0.16, 0.42]	14	0.30 [0.21, 0.42]	10	-
**Total flavonoids**	1.46		0.82		1.52		
**Total phenolic compounds**	164.65		121.60		138.22		

* Values represent the median concentration with the interquartile range [Q1–Q3]. Within a row, values with different superscript letters (a, b) are significantly different (*p* < 0.05), based on the Kruskal–Wallis test followed by a Conover post hoc analysis. Statistical testing was not performed for compounds where a value could not be determined in 10 or more samples (e.g., pyrogallol, rutin, chrysin), hence no *p*-value is provided. Abbreviations: HGA = Homogentisic acid; DBA34 = 3,4-Dihydroxybenzoic acid (protocatechuic acid); DB34 = 3,4-dihydroxy-benzaldehyde; p-CA = p-coumaric acid; t-CA = Trans-cinnamic acid; and ND = Not detectable.

**Table 4:** Comparative performance metrics for supervised classification models based on phenolic compound profiles.

Methods	Class (Region)	Predicted			Total	Accuracy	Precision (PPV)	Recall (TPR)	False Positive Rate (FPR)	False Discovery Rate (FDR)	F1 Score	Matthews Correlation Coefficient (MCC)	Negative Predictive Value (NPV)	True Negative Rate (TNR)	False Negative Rate (FNR)	AUC
		Şırnak Faraşin	Siirt Merkez	Siirt Pervari												
PLS-DA	Şırnak Faraşin	12	5	0	17	0.824	0.750	0.706	0.118	0.250	0.727	0.598	0.857	0.882	0.294	0.706
	Siirt Merkez	3	11	4	18	0.569	0.423	0.611	0.455	0.577	0.500	0.150	0.720	0.545	0.389	0.599
	Siirt Pervari	1	10	5	16	0.706	0.556	0.313	0.114	0.444	0.400	0.241	0.738	0.886	0.688	0.584
	Total	16	26	9	51	0.549	0.576	0.543	0.229	0.424	0.542	0.330	0.772	0.771	0.457	0.630
Random Forest	Şırnak Faraşin	13	3	1	17	0.882	0.765	0.765	0.118	0.235	0.765	0.647	0.882	0.882	0.235	0.851
	Siirt Merkez	2	10	6	18	0.647	0.500	0.556	0.303	0.500	0.526	0.247	0.742	0.697	0.444	0.657
	Siirt Pervari	2	7	7	16	0.686	0.500	0.438	0.200	0.500	0.467	0.247	0.757	0.800	0.563	0.731
	Total	17	20	14	51	0.588	0.588	0.586	0.207	0.412	0.586	0.380	0.794	0.793	0.414	0.746
SVM	Şırnak Faraşin	12	3	2	17	0.804	0.706	0.706	0.147	0.294	0.706	0.559	0.853	0.853	0.294	0.787
	Siirt Merkez	2	11	5	18	0.647	0.500	0.611	0.333	0.500	0.550	0.268	0.759	0.667	0.389	0.522
	Siirt Pervari	3	8	5	16	0.647	0.417	0.313	0.200	0.583	0.357	0.123	0.718	0.800	0.688	0.593
	Total	17	22	12	51	0.549	0.541	0.543	0.227	0.459	0.538	0.317	0.777	0.773	0.457	0.634

## Data Availability

The original contributions presented in the study are included in the article/Supplementary Materials, further inquiries can be directed to the corresponding author.

## Supplementary Materials

The following supporting information can be downloaded at https://www.mdpi.com/article/10.3390/foods14173105/s1. Supplementary Material S1. Methods Parameters. Supplementary Material S2. PCA_Loadings of Variables. Supplementary Material S3. Supervised Methods Data. Supplementary Materials S4. Percentage Contribution of Phenolic Compounds.

## References

[1] Bogdanov S., Jurendic T., Sieber R., Gallmann P. (2008). Honey for Nutrition and Health: A Review. J. Am. Coll. Nutr..

[2] Kuś P.M. (2020). Honey as Source of Nitrogen Compounds: Aromatic Amino Acids, Free Nucleosides and Their Derivatives. Molecules.

[3] Brugnerotto P., Fuente-Ballesteros A., Martín-Gómez B., Ares A.M., Gonzaga L.V., Fett R., Costa A.C.O., Bernal J. (2024). Free amino acid profile in Mimosa scabrella honeydew honey from Brazil and chemometric analysis for geographical discrimination. Food Res. Int..

[4] Rebane R., Herodes K. (2008). Evaluation of the botanical origin of Estonian uni- and polyfloral honeys by amino acid content. J. Agric. Food Chem..

[5] Anklam E. (1998). A review of the analytical methods to determine the geographical and botanical origin of honey. Food Chem..

[6] Alvarez-Suarez A.J.M., Giampieri F., Brenciani A., Mazzoni L., Gasparrini M., González-Paramás A.M., Santos-Buelga C., Morroni G., Simoni S., Forbes-Hernández T.Y. (2018). Apis mellifera vs Melipona beecheii Cuban polifloral honeys: A comparison based on their physicochemical parameters, chemical composition and biological properties. LWT.

[7] Everstine K.D., Chin H.B., Lopes F.A., Moore J.C. (2024). Database of Food Fraud Records: Summary of Data from 1980 to 2022. J. Food Prot..

[8] Geana E.I., Ciucure C.T. (2020). Establishing authenticity of honey via comprehensive Romanian honey analysis. Food Chem..

[9] Soares S., Amaral J.S., Oliveira M.B.P.P., Mafra I.A. (2017). Comprehensive Review on the Main Honey Authentication Issues: Production and Origin. Compr. Rev. Food Sci. Food Saf..

[10] European Commission Honey Market Presentation. https://agriculture.ec.europa.eu/document/download/c04a9774-5ba3-41f5-b256-08396b2888ec_en?filename=market-presentation-honey_spring2024_en.pdf.

[11] Jensen J.D., Mørkbak M.R. (2013). The role of gastronomic, externality, and feasibility attributes in consumer demand for organic and local foods: The case of honey and apples. Int. J. Consum. Stud..

[12] Cosmina M., Gallenti G., Marangon F., Troiano S. (2016). Reprint of “Attitudes towards honey among Italian consumers: A choice experiment approach”. Appetite.

[13] Tsagkaris A.S., Koulis G.A., Danezis G.P., Martakos I., Dasenaki M., Georgiou C.A., Thomaidis N.S. (2021). Honey authenticity: Analytical techniques, state of the art and challenges. RSC Adv..

[14] Ballco P., Jaafer F., de Magistris T. (2022). Investigating the price effects of honey quality attributes in a European country: Evidence from a hedonic price approach. Agribusiness.

[15] Wang J., Li Q.X., Taylor S.L. (2011). Chapter 3. Chemical Composition, Characterization, and Differentiation of Honey Botanical and Geographical Origins. Advances in Food and Nutrition Research.

[16] Vazquez L., Armada D., Celeiro M., Dagnac T., Llompart M. (2021). Evaluating the Presence and Contents of Phytochemicals in Honey Samples: Phenolic Compounds as Indicators to Identify Their Botanical Origin. Foods.

[17] She S., Chen L., Song H., Lin G., Li Y., Zhou J., Liu C. (2019). Discrimination of geographical origins of Chinese acacia honey using complex 13C/12C, oligosaccharides and polyphenols. Food Chem..

[18] Nyarko K., Boozer K., Greenlief C.M. (2023). Profiling of the polyphenol content of honey from different geographical origins in the United States. Molecules.

[19] Cheung Y., Meenu M., Yu X., Xu B. (2019). Phenolic acids and flavonoids profiles of commercial honey from different floral sources and geographic sources. Int. J. Food Prop..

[20] Iurlina M.O., Saiz A.I., Fritz R., Manrique G.D. (2009). Major flavonoids of Argentinean honeys: Optimisation of the extraction method and analysis of their content in relationship to the geographical source of honeys. Food Chem..

[21] Cometto P.M., Faye P.F., Di Paola Naranjo R.D., Rubio M.A., Aldao M.A. (2003). Comparison of free amino acids profile in honey from three Argentinian regions. J. Agric. Food Chem..

[22] Ren W., Li Y., Yin Y., Blachier F., Blachier F., Wu G., Yin Y. (2013). Overview. Nutritional and Physiological Functions of Amino Acids in Pigs.

[23] Davies A.M.C. (1975). Amino acid analysis of honeys from eleven countries. J. Apic. Res..

[24] Yao L., Datta N., Tomás-Barberán F.A., Ferreres F., Martos I., Singanusong R. (2003). Flavonoids, phenolic acids and abscisic acid in Australian and New Zealand Leptospermum honeys. Food Chem..

[25] Silva T.M.S., dos Santos F.P., Evangelista-Rodrigues A., da Silva E.M.S., da Silva G.S., de Novais J.S., dos Santos F.A.R., Camara C.A. (2013). Phenolic compounds, melissopalynological, physicochemical analysis and antioxidant activity of jandaíra (*Melipona subnitida*) honey. J. Food Compos. Anal..

[26] Silici S., Sagdic O., Ekici L. (2010). Evaluation of the phenolic content, antiradical, and antimicrobial activity of Rhododendron honeys. Food Chem..

[27] Aljadi A.M., Kamaruddin M.Y. (2004). Evaluation of the phenolic contents and antioxidant capacities of two Malaysian floral honeys. Food Chem..

[28] Ulusoy E., Kolaylı S., Sarıkaya A.O. (2010). Antioxidant and antimicrobial activity of different floral origin honeys from Türkiye. J. Food Biochem..

[29] Cianciosi D., Forbes-Hernández T.Y., Afrin S., Gasparrini M., Reboredo-Rodriguez P., Manna P.P., Zhang J., Bravo Lamas L., Martínez Flórez S., Agudo Toyos P. (2018). Phenolic compounds in honey and their associated health benefits: A review. Molecules.

[30] Abedi F., Razavi B.M., Hosseinzadeh H. (2019). A review on gentisic acid as a plant-derived phenolic acid and metabolite of aspirin: Comprehensive pharmacology, toxicology, and some pharmaceutical aspects. Phytother. Res..

[31] Feng G., Zhang L., Bao W., Ni J., Wang Y., Huang Y., Lyv J., Cao X., Chen T., You K. (2024). Gentisic acid prevents colorectal cancer metastasis via blocking GPR81-mediated DEPDC5 degradation. Phytomedicine.

[32] Picone G., Mezzetti B., Babini E., Capocasa F., Placucci G., Capozzi F. (2011). Unsupervised principal component analysis of NMR metabolic profiles for the assessment of substantial equivalence of transgenic grapes (*Vitis vinifera*). J. Agric. Food Chem..

[33] Kivrak İ. (2015). Free Amino Acid Profiles of 17 Turkish Unifloral Honeys. J. Liq. Chromatogr. Relat. Technol..

[34] Thiele B., Stein N., Oldiges M., Hofmann D. (2012). Direct analysis of underivatized amino acids in plant extracts by LC-MS-MS. Amino Acid Analysis.

[35] Kowalski S., Kopuncová M., Ciesarová Z., Kukurová K. (2017). Free amino acids profile of Polish and Slovak honeys based on LC–MS/MS method without the prior derivatisation. J. Food Sci. Technol..

[36] Kıvrak Ş., Kıvrak İ. (2017). Assessment of phenolic profile of Turkish honeys. Int. J. Food Prop..

[37] Davies A.M.C. (1976). The application of amino acid analysis to the determination of the geographic origin of honey. J. Food Technol..

[38] Davies A.M.C., Harris R.G. (1982). Free Amino Acid Analysis of Honeys from England and Wales: Application to the Determination of the Geographical Origin of Honeys. J. Apic. Res..

[39] Gilbert J., Shephard M.J., Wallwork M.A., Harris R.G. (1981). Determination of the geographical origin of honeys by multivariate analysis of gas chromatographic data on their free amino acid content. J. Apic. Res..

[40] Cesare Marincola F., Palmas C., Lastres Couto M.A., Paz I., Cremades J., Pintado J., Bruni L., Picone G. (2025). Metabolic Profile of Senegalese Sole (Solea senegalensis) Muscle: Effect of Fish–Macroalgae IMTA-RAS Aquaculture. Molecules.

[41] Valverde S., Ares A.M., Elmore J.S., Bernal J. (2022). Recent trends in the analysis of honey constituents. Food Chem..

[42] Cotte J.F., Casabianca H., Giroud B., Albert M., Lheritier J., Grenier-Loustalot M.F. (2004). Characterization of honey amino acid profiles using high-pressure liquid chromatography to control authenticity. Anal. Bioanal. Chem..

[43] Chen H., Jin L., Chang Q., Peng T., Hu X., Fan C., Pang G., Wang W. (2017). Discrimination of botanical origins for Chinese honey according to free amino acids content by high-performance liquid chromatography with fluorescence detection with chemometric approaches. J. Sci. Food Agric..

[44] Hermosín I., Chicón R.M., Cabezudo M.D. (2003). Free amino acid composition and botanical origin of honey. Food Chem..

[45] Iglesias M.T., De Lorenzo C., Del Carmen Polo M., Martín-Alvarez P.J., Pueyo E. (2004). Usefulness of amino acid composition to discriminate between honeydew and floral honeys. Application to honeys froma small geographic area. J. Agric. Food Chem..

[46] Food and Agriculture Organisation of the United Nations Crops and Livestock Products. https://www.fao.org/faostat/en/#data/QCL.

[47] Republic of Turkey Ministry of Enviroment and Forestry UN Convention of Biological Diversity Fourth National Report 30/06/2009. https://www.cbd.int/doc/world/tr/tr-nr-04-en.pdf.

[48] Can Z., Yildiz O., Sahin H., Akyuz Turumtay E., Silici S., Kolayli S. (2015). An investigation of Turkish honeys: Their physicochemical properties, antioxidant capacities and phenolic profiles. Food Chem..

[49] Şenyuva H.Z., Gilbert J., Silici S., Charlton A., Dal C., Gürel N., Cimen D. (2009). Profiling Turkish Honeys to Determine Authenticity Using Physical and Chemical Characteristics. J. Agric. Food Chem..

[50] Silici S., Karaman K. (2014). Chemometric approaches for the characterization of turkish rhododendron and honeydew honeys depending on amino acid composition. J. Liq. Chromatogr. Relat. Technol..

[51] Turkish Agriculture and Forestry Journal. http://www.turktarim.gov.tr/Haber/761/siirtten-sifa-kaynagi-pervari-bali.

[52] UNDP Türkiye Organic Agriculture Cluster. https://www.undp.org/turkiye/projects/organic-agriculture-cluster.

[53] Republic of Türkiye Ministry of Industry and Technology Başarılar: Arıcılık. https://www.gap.gov.tr/sayfa/basari-hikayeleri/basarilar/aricilik/.

[54] Republic of Türkiye Ministry of Agriculture and Forestry Apiculture Research Institute (2024). Beekeeping Statistics by Province in Türkiye. https://arastirma.tarimorman.gov.tr/aricilik/Link/2/Aricilik-Istatistikleri.

[55] Turkish Patent and Trademark Office Official Statistics. https://ci.turkpatent.gov.tr/cografi-isaretler/detay/37928.

[56] Turkish Patent and Trademark Office Official Statistics. https://ci.turkpatent.gov.tr/cografi-isaretler/detay/8563.

[57] Gürbüz S., Özenirler Ç., Mayda N., Gençay Çelemli Ö., Özkök A. (2019). Pollen Spectrum of Some Honey Samples Produced in Siirt-Turkey. Hacet. J. Biol. Chem..

[58] Gürbüz S., Gençay Çelemli Ö., Özenirler Ç., Mayda N., Özkök A., Sorkun K. (2019). Melissopalnological analysis of honey samples collected from Şirnak City. Uludag Bee J..

[59] Kıvrak İ., Kıvrak Ş., Harmandar M. (2014). Free Amino Acid Profiling in the Giant Puffball Mushroom (*Calvatia gigantea*) Using UPLC-MS/MS. Food Chem..

[60] Azevedo M.S., Seraglio S.K.T., Rocha G., Balderas C.B., Piovezan M., Gonzaga L.V., Falkenberg D.D.B., Fett R., de Oliveira M.A.L., Costa A.C.O. (2017). Free amino acid determination by GC-MS combined with a chemometric approach for geographical classification of bracatinga honeydew honey (*Mimosa scabrella* Bentham). Food Control.

[61] Łozowicka B., Kaczyńsk P., Iwaniuk P. (2021). Analysis of 22 free amino acids in honey from Eastern Europe and Central Asia using LC-MS/MS technique without derivatization step. J. Food Compos. Anal..

[62] Yang J., Liu Y., Cui Z., Wang T., Liu T., Liu G. (2024). Analysis of Free Amino Acid Composition and Honey Plant Species in Seven Honey Species in China. Foods.

[63] Conte L.S., Miorini M., Giomo A., Bertacco G., Zironi R. (1998). Evaluation of some fixed components for unifloral honey characterization. J. Agric. Food Chem..

[64] Maione C., Barbosa F., Melgaço Barbosa R. (2019). Predicting the botanical and geographical origin of honey with multivariate data analysis and machine learning techniques: A review. Comput. Electron. Agric..

[65] Satari A., Ghasemi S., Habtemariam S., Asgharian S., Lorigooini Z. (2021). Rutin: A flavonoid as an effective sensitizer for anticancer therapy; insights into multifaceted mechanisms and applicability for combination therapy. Evid.-Based Complement. Altern. Med..

[66] Moniruzzaman M., Sulaiman S.A., Gan S.H. (2017). Phenolic acid and flavonoid composition of Malaysian honeys. J. Food Biochem..

[67] Ganai A.A., Farooqi H. (2015). Bioactivity of genistein: A review of in vitro and in vivo studies. Biomed. Pharmacother..

