# Supplementary material for: Chemometric Differentiation of Organic Honeys from Southeastern Türkiye Based on Free Amino Acid and Phenolic Profiles
Source: Foods. 2025 Sep 5;14(17):3105. doi: 10.3390/foods14173105 (PMC12428088; doi:10.3390/foods14173105)
Supplement: Supplementary file 1 [file foods-14-03105-s001.zip › Supplementary Material-S1-Methods Parameters_seyda.pdf]

Supplementary Materials (S1)

Table S1. Chromatographic and MRM method parameters for free amino acids using UPLC–MS/MS.

**Amino acid**	***t*** t_R **(min)**[a]	**Quantification transition (*m/z*)**	**Confirmatory transition (*m/z*)**	**CE (V)**[b]
Glycine	0.58	76.00	30.00, 44.00, 76.00	8, 8, 3
Alanine	0.59	90.00	57.10, 71.00	8, 8
Serine	0.58	106.00	60.00, 88.00	9, 10
Proline	0.67	116.10	43.30, 70.10	22, 12
Valine	0.83	118.10	55.00, 72.00	18, 10
Threonine	0.61	120.10	56.10, 74.00, 84.00, 102.10	15, 10, 12, 9
Leucine	1.67	132.10	69.20, 86.00	20, 10
Isoleucine	1.55	132.20	69.20, 86.10	20, 9
Asparagine	0.58	133.10	74.00, 87.13, 115.10	15, 10, 10
Aspartic acid	0.59	134.10	74.00, 88.00, 116.00	14, 10, 8
Lysine	0.59	147.00	84.00, 115.00, 130.10	20, 12, 10
Glutamine	0.59	147.10	84.10, 130.10	16, 10
Glutamic acid	0.60	148.10	84.00, 102.10, 130.20	15, 12, 8
Methionine	0.99	150.20	56.10, 104.10, 133.20	15, 10, 9
Histidine	0.56	156.10	83.10, 93.10, 110.19	22, 20, 15
Phenylalanine	3.41	166.20	77.00, 91.20,103.10,120.00	30, 30, 25, 14
Arginin	0.57	175.20	60.00, 70.00, 116.00	15, 20, 15
Tyrosine	1.35	182.16	123.10, 136.10, 165.06	15, 15, 9
Tryptophan	4.25	205.10	91.00, 118.10, 188.16	35, 25, 10
Cysteine	0.65	241.30	74.00, 120.00, 152.00	25, 20, 12

[a] t_R: retention time
[b] CE (V): collision energies

Table S2. Chromatographic Conditions for UPLC-ESI-MS/MS Analysis of Free Amino Acids

Time (min)	Flow Rate (mL/min)	Mobil Phase A (%)	Mobil Phase B (%)
0.00	0.400	100.00	00.00
2.00	0.400	100.00	00.00
8.00	0.400	30.00	70.00
9.00	0.400	100.00	00.00
10.00	0.400	100.00	00.00

Table S3. LoD values, recovery, the regression equations, and correlation coefficients of phenolic compounds.

Compounds	LoD (mg/kg)	Recovery (%)	R^2	Calibration equations
Genistein	0.020	80.0–89.1	0.949010	$y = 7432.24x - 51893.9$
Galanthamine	0.010	92.1–100.1	0.940432	$y = 3016.64x + 186550$
Quercetin	0.009	84.5–103.1	0.999425	$y = 144.773x - 3372.83$
Pyrocatechol	0.010	100.3–105.7	0.978938	$y = 26.558x - 695.012$
Pyrogallol	0.010	99.7–100.2	0.944105	$y = 12.1362x - 838.226$
4-hydroxy benzoic acid	0.019	85.7–99.6	0.950047	$y = 150.172x - 5788.02$
3,4-dihydroxy benzaldehyde	0.015	97.3–101.4	0.979163	$y = 33.2807x - 1357.91$
trans-Cinnamic acid	0.010	94.4–98.0	0.945931	$y = 483.633x + 20579.6$
Vanillin	0.010	91.3–94.9	0.989117	$y = 716.252x + 13260.9$
Gentisic acid	0.020	98.9–99.9	0.999086	$y = 23.5686x + 85.0228$
3,4-dihydroxybenzoic acid	0.019	99.1–100.9	0.967247	$y = 40.023x + 1692.01$
p-Coumaric acid	0.018	88.0–100.1	0.922654	$y = 124.038x - 10356.7$
trans-2-hydroxy cinnamic acid	0.010	98.1–99.9	0.924930	$y = 104.604x - 5575.01$
Vanillic acid	0.010	97.9–99.9	0.959589	$y = 8.65344x - 531.706$
Homogentisic acid	0.011	90.2–93.7	0.998897	$y = 2.33669x - 59.969$
Gallic acid	0.010	98.6–100.8	0.966953	$y = 25.3523x + 69.1892$
Caffeic acid	0.010	89.9–99.1	0.993233	$y = 96.8219x + 2457.26$
Ferulic acid	0.012	87.4–96.8	0.926328	$y = 3.35506x + 124.486$
Syringic acid	0.010	85.1–93.4	0.954535	$y = 3.66469x + 312.343$
Resveratrol	0.009	85.5–108.7	0.987002	$y = 227.114x + 3190.96$
Chrysin	0.010	98.7–100.5	0.961230	$y = 527.767x - 8248.27$
Apigenin	0.009	100.7–108.9	0.926277	$y = 643.035x + 18526.3$
Naringenin	0.010	96.1–99.6	0.944934	$y = 399.76x - 3760.69$
Kaempferol	0.011	94.1–96.9	0.960047	$y = 53.7117x - 1349.26$
Luteolin	0.010	95.4–98.7	0.991112	$y = 158.435x - 3330.72$
Catechin hydrate	0.010	98.3–101.7	0.924853	$y = 31.2824x - 250.631$
Epicatechin	0.010	96.8–100.6	0.966490	$y = 32.4033x + 1338.53$
Hesperetin	0.011	90.0–94.1	0.980921	$y = 906.251x - 20648.3$
Myricetin	0.010	94.5–101.6	0.936414	$y = 0.289311x + 7.70349$
Catechin gallate	0.010	96.1–100.0	0.954381	$y = 14.2448x - 821.038$

Rutin	0.009	97.2–103.6	0.993205	$y = 72.6334x + 5524.59$
Chlorogenic acid	0.010	91.5–93.4	0.911208	$y = 1.06677x + 33.37$
