# Supplementary material for: Chemometric Differentiation of Organic Honeys from Southeastern Türkiye Based on Free Amino Acid and Phenolic Profiles
Source: Foods. 2025 Sep 5;14(17):3105. doi: 10.3390/foods14173105 (PMC12428088; doi:10.3390/foods14173105)
Supplement: Supplementary file 1 [file foods-14-03105-s001.zip › Supplementary Materials-S4-Percentage Contribution of Phenolic Compounds.pdf]

Supplementary Materials-4
Percentage Contribution of Phenolic Compounds

Table S1. Percentage contribution of individual phenolic acids to the total phenolic acid content in honeys from the three geographical regions.

Phenolic Acid	Şırnak Faraşin (%)	Siirt Merkez (%)	Siirt Pervari (%)
Pyrogallol	0.42	0.55	0.90
HGA	1.92	2.33	1.17
DBA34	6.25	3.34	1.98
Gentisic acid	61.65	47.61	48.65
DB34	0.86	0.56	1.36
Vanilic acid	5.22	8.86	7.19
Caffeic acid	18.38	24.42	23.77
Vanillin	0.03	0.02	0.05
p-CA	5.23	12.24	14.85
t-CA	0.03	0.07	0.07

Table S2. Percentage contribution of individual flavonoids to the total flavonoid content in honeys from the three geographical regions.

Flavonoids	Şırnak Faraşin (%)	Siirt Merkez (%)	Siirt Pervari (%)
Rutin	39.73	20.73	19.74
Luteolin	23.97	26.83	36.18
Quercetin	9.59	8.54	7.89
Genistein	9.59	14.63	9.87
Hesperetin	9.59	0,00	6.58
Chrysin	7.53	29.27	19.74
