# Supplementary material for: Chemometric Differentiation of Organic Honeys from Southeastern Türkiye Based on Free Amino Acid and Phenolic Profiles
Source: Foods. 2025 Sep 5;14(17):3105. doi: 10.3390/foods14173105 (PMC12428088; doi:10.3390/foods14173105)
Supplement: Supplementary file 1 [file foods-14-03105-s001.zip › Supplementary Material_S2-PCA_Loadings of Variables.pdf]

Supplementary Materials-2

PCA DATA

Table S1. Loading Values of Amino Acid Components as a Result of Principal Component Analysis (PCA)

FAA	PCA1	PCA2	PCA3	PCA4	PCA5	PCA6	PCA7
Gly	0.544	-0.052	-0.279	-0.025	-0.188	0.106	-0.140
Ala	0.371	0.044	-0.527	-0.031	0.095	0.472	0.304
Ser	0.381	0.251	0.464	-0.195	0.526	0.076	-0.232
Pro	-0.006	0.161	0.506	0.022	-0.127	0.668	-0.178
Val	0.031	0.616	-0.019	-0.158	0.312	-0.294	0.203
Thr	-0.018	0.612	0.150	0.407	-0.169	0.137	0.451
Cys	0.640	-0.413	0.114	0.154	-0.022	0.151	0.071
Leu	0.135	0.113	-0.516	0.430	0.368	-0.084	-0.273
Ile	0.158	0.639	0.182	0.401	0.231	0.062	0.076
Asn	0.189	0.644	-0.226	-0.065	-0.432	-0.153	0.113
Asp	0.048	-0.508	-0.058	0.493	0.055	-0.063	-0.087
Lys	-0.321	-0.126	-0.306	-0.567	0.255	0.264	0.259
Gln	0.779	-0.047	-0.158	-0.214	-0.076	-0.128	-0.117
Glu	0.731	-0.029	-0.002	-0.025	0.010	0.256	0.017
Met	0.776	0.051	-0.017	0.099	-0.307	-0.097	-0.003
His	0.720	-0.102	0.106	-0.230	0.061	-0.116	0.301
Phe	0.725	-0.157	0.209	-0.050	-0.130	-0.091	-0.041
Arg	0.520	-0.273	-0.082	0.263	0.416	0.004	0.268
Tyr	-0.093	-0.551	0.440	0.043	0.009	-0.252	0.451
Trp	0.587	0.368	0.148	-0.212	0.114	-0.192	-0.180
eigenvalue	4.585	2.682	1.613	1.397	1.214	1.137	1.028
percentage of variance	22.926	13.410	8.066	6.986	6.072	5.683	5.142
cumulative							

percentage of variance	22.926	36.336	44.402	51.388	57.460	63.142	68.284

Table S2. Phenolic Acid PCA data

	eigenvalue	percentage of variance	cumulative percentage of variance
comp 1	5.424	54.238	54.238
comp 2	1.134	11.345	65.583
comp 3	1.063	10.633	76.216
comp 4	0.625	6.248	82.464
comp 5	0.533	5.331	87.795
comp 6	0.411	4.111	91.906
comp 7	0.313	3.128	95.034
comp 8	0.243	2.431	97.464
comp 9	0.153	1.530	98.994
comp 10	0.101	1.006	100.000

Table S3. Flavonoid PCA data

	eigenvalue	percentage of variance	cumulative percentage of variance
comp 1	3.300	54.995	54.995
comp 2	0.934	15.558	70.553
comp 3	0.695	11.579	82.132
comp 4	0.582	9.693	91.825
comp 5	0.364	6.068	97.893
comp 6	0.126	2.107	100.000

Table S4. Phenolic Acid and Flavonoid PCA data

	eigenvalue	percentage of variance	cumulative percentage of variance
comp 1	8.323	52.019	52.019
comp 2	1.585	9.904	61.923
comp 3	1.335	8.345	70.268
comp 4	0.962	6.011	76.278
comp 5	0.740	4.627	80.905
comp 6	0.636	3.977	84.882
comp 7	0.471	2.946	87.828
comp 8	0.469	2.934	90.762
comp 9	0.385	2.409	93.170
comp 10	0.305	1.904	95.075
comp 11	0.213	1.334	96.409
comp 12	0.200	1.247	97.656
comp 13	0.152	0.947	98.603
comp 14	0.095	0.596	99.200
comp 15	0.080	0.499	99.699
comp 16	0.048	0.301	100.000
