# Supplementary material for: Chemometric Differentiation of Organic Honeys from Southeastern Türkiye Based on Free Amino Acid and Phenolic Profiles
Source: Foods. 2025 Sep 5;14(17):3105. doi: 10.3390/foods14173105 (PMC12428088; doi:10.3390/foods14173105)
Supplement: Supplementary file 1 [file foods-14-03105-s001.zip › Supplementary Material_S3-Supervised Methods Data.pdf]

Supplementary Materials 3
Supervised Methods

PLS-DA METHOD:

Calculation Details:

Following the process outlined in the Materials and Methods section of the article, the optimal number of components was determined as 2, and the final model was built with these parameters. For method performance, the confusion matrix, overall accuracy rate, and Precision, Recall, F1 score, TNR, FNR, and MCC values for each class are reported. In addition, one-vs-rest ROC curves and AUC values obtained from the class-based score matrices were calculated. The contribution of the variables to the model was assessed using PLS-DA component loadings, and the amino acids with the highest loading values for the first two components (Comp1: 19%, Comp2: 14% of the variance explained) are presented in the table. These results indicate that the relevant amino acids play a decisive role in distinguishing between classes. A fixed seed (seed = 123) was used in all analyses to reduce the effect of randomness.

Table S1. PLS-DA Component Loadings Table for Amino Acid Components

Variable (Amino Acid)	Component 1 (19% expl. variance)	Component 2 (14% expl. variance)
Tyr	2.231	1.944
Glu	1.317	1.043
Met	1.168	1.031
Gln	1.168	0.967
Ser	1.074	0.869
Asn	1.008	1.016
Val	0.995	0.794
His	0.993	1.494
Gly	0.988	1.165
Ile	0.975	0.799
Trp	0.860	0.816
Ala	0.807	0.602
Pro	0.766	0.796
Phe	0.761	0.749
Arg	0.736	1.424
Asp	0.695	0.938
Leu	0.669	0.693
Thr	0.656	0.509

Cys	0.169	0.780
Lys	0.027	0.095

When the component loadings obtained as a result of PLS-DA analysis are examined, the contribution levels of amino acids on the first component (19% explained variance) and the second component (14% explained variance) differ. The highest loading values in the first component were found to be tyrosine (Tyr; 2.231), glutamic acid (Glu; 1.317), methionine (Met; 1.168), and glutamine (Gln; 1.168), respectively. In the second component, the highest loadings were determined for tyrosine (Tyr; 1.944), histidine (His; 1.494), arginine (Arg; 1.424), and glycine (Gly; 1.165). These amino acids stand out as the source of significant variation among the different groups on the second component. Lower loading values (e.g., lysine [Lys; 0.027 and 0.095]) show limited discrimination in these components.

Table S2. PLS-DA Component Loadings Table for Phenolic Compounds

Variable (Phenolic Compound)	Component 1 (13% expl. variance)	Component 2 (44% expl. variance)
p-CA	2.208	2.047
Hesperetin	2.027	1.936
Chrysin	1.424	1.346

t-CA	1.091	1.074
Gentisic acid	0.910	0.916
HGA	0.857	0.796
Pyrogallol	0.704	0.835
Vanillic acid	0.604	0.565
Rutin	0.590	0.811
DBA34	0.548	0.526
Quercetin	0.525	0.672
DB34	0.453	0.431
Luteolin	0.424	0.613
Vanillin	0.221	0.452
Genistein	0.095	0.647
Caffeic acid	0.060	0.194

When the PLS-DA component loadings of the phenolic acid components are examined, the first component (comp1) explains 13% of the total variance, while the second component (comp2) explains 44%. The compounds with the highest loading values in the first component are p-coumaric acid (p-CA; 2.208), hesperetin (2.027), and chrysin (1.424), which stand out as the elements with the strongest discriminatory properties in the first component of the model. t-CA (1.091), gentisic acid (0.910), and HGA (0.857) also stand out with their high loading values.

In the second component, p-CA (2.047) and hesperetin (1.936) also have the highest loading values, demonstrating a common and strong discriminatory property across both components.

Random Forest Method

Calculation Details:

Following the process outlined in the Materials and Methods section of the article, the maximum depth was not further constrained, and default packet stopping criteria (e.g., at least one observation in the terminal node, nodesize=1) were used. Method performance was calculated using out-of-fold (OOF) estimates, and class-based metrics and AUC values were reported. Furthermore, the significance of the variables was calculated, and the variables with the highest contribution were identified.

Table S3. Importance Levels of Amino Acids in the Random Forest

Variable (Amino Acid)	Şırnak Faraşin	Siirt Merkez	Siirt Pervari	Overall
Tyr	100.00	69.37	46.81	72.06
His	4.53	41.92	54.74	33.73
Arg	10.74	44.64	38.57	31.31
Met	30.82	22.23	19.70	24.25
Trp	32.65	26.20	12.63	23.83
Glu	29.69	6.67	32.98	23.12
Ile	32.29	23.60	2.17	19.35
Gln	18.19	6.21	28.51	17.64
Asp	27.79	18.87	6.10	17.59
Gly	13.39	4.36	32.69	16.82
Asn	34.90	9.03	5.08	16.34
Cys	5.47	16.40	18.48	13.45
Leu	15.56	14.96	6.40	12.31
Pro	13.28	8.83	11.20	11.10
Phe	5.30	14.56	11.76	10.54
Lys	11.12	1.70	18.10	10.31
Val	15.66	4.52	10.69	10.29
Ala	19.27	9.56	0.00	9.61

Ser	15.07	6.08	6.54	9.23
Thr	12.30	2.11	1.86	5.42

Using the Random Forest classification model, the contribution of amino acids to the classification performance was evaluated based on their importance percentages. The results show that amino acids that play a significant role in the discrimination of different groups (Şırnak Faraşin, Siirt Merkez, Siirt Pervari) stand out. In the Şırnak Faraşin group, tyrosine (Tyr; 100.00%) provided the highest contribution to the classification, followed by asparagine (Asn; 34.90%) and tryptophan (Trp; 32.65%). In the Siirt Merkez group, histidine (His; 41.92%) and arginine (Arg; 44.64%) stood out with high importance scores. In the Siirt Pervari group, histidine (His; 54.74%) and glycine (Gly; 32.69%) were found to be the most significant distinguishing features. When looking at overall significance percentages, the highest values belong to the amino acids tyrosine (Tyr; 72.06%), histidine (His; 33.73%), arginine (Arg; 31.31%), and methionine (Met; 24.25%). These results reveal that these amino acids are the biochemical markers most frequently used by the

model to distinguish between the three groups. Variables with lower significance scores (e.g., threonine [Thr; 5.42%]) were considered to carry relatively less information in the classification.

Table S4. Importance Levels of Phenolic Compounds in the Random Forest

Variable (Phenolic Compound)	Şırnak Faraşin	Siirt Merkez	Siirt Pervari	Overall
Hesperetin	100.00	87.64	41.97	76.54
Luteolin	38.69	64.13	78.12	60.31
DBA34	73.52	37.43	67.99	59.65
p-CA	74.44	10.56	35.61	40.20
Chrysin	55.11	17.47	41.08	37.89
HGA	20.24	31.56	54.30	35.37
Vanillic acid	60.78	26.81	17.83	35.14
DB34	27.40	34.22	37.90	33.17
Gentisic acid	1.50	51.29	27.19	26.66
Quercetin	22.72	39.60	17.02	26.45
Genistein	28.55	27.56	17.49	24.53
t-CA	23.49	9.65	33.98	22.37
Rutin	25.47	7.22	29.73	20.80
Pyrogallol	15.80	7.86	32.39	18.68

Caffeic acid	22.89	4.83	27.43	18.38
Vanillin	11.68	0.00	24.60	12.09

When the contribution of phenolic acid compounds to the classification success was examined in the Random Forest analysis, hesperetin was determined to be the compound with the highest significance across all regions (Şırnak Faraşin: 100.00; Siirt Center: 87.64; Siirt Pervari: 41.97; overall significance: 76.54). Luteolin (60.31% overall significance) and DBA34 (59.65%) were other highly significant compounds.

Regionally, hesperetin (100%), p-CA (74.44%), and DBA34 (73.52%) were prominent in the Şırnak Faraşin samples, while hesperetin (87.64%), luteolin (64.13%), and gentisic acid (51.29%) were the most significant compounds in Siirt Center. In Siirt Pervari samples, luteolin (78.12%), DBA34 (67.99%) and HGA (54.30%) attracted attention with their high significance levels.

SVM Method

Calculation Details:

Following the process outlined in the Materials and Methods section of the article, the method's performance was evaluated using OOF predictions; confusion matrix, accuracy, class-based metrics (Precision, Recall, F1, MCC, TNR, FNR), and AUC values were reported. Variable importance ranking was calculated using the varImp function of the caret package.

Table S5. Importance Levels of Amino Acids in the SVM

Variable (Amino Acid)	Şırnak Faraşin	Siirt Merkez	Siirt Pervari	Overall
Tyr	100.00	100.00	98.56	99.52
His	51.44	76.03	76.03	67.84
Arg	38.04	67.05	67.05	57.38
Glu	65.27	31.12	65.27	53.89

Gly	47.12	49.09	49.09	48.43
Trp	48.41	46.21	48.41	47.68
Asn	48.51	48.51	41.50	46.17
Asp	57.73	57.73	20.92	45.46
Gln	54.47	26.22	54.47	45.05
Ile	43.13	43.13	31.99	39.42
Ser	46.69	22.14	46.69	38.50
Met	45.39	21.33	45.39	37.37
Val	38.52	38.52	31.99	36.34
Phe	37.18	27.04	37.18	33.80
Ala	38.90	17.39	38.90	31.73
Cys	28.15	28.15	22.96	26.42
Pro	23.15	23.15	20.75	22.35
Leu	25.46	25.46	11.24	20.72
Thr	18.92	18.92	16.43	18.09
Lys	0.00	7.44	7.44	4.96

According to the results of the variable importance analysis conducted with Support Vector Machines (SVM), Tyr was the variable with the highest discriminatory power in the classification of Şırnak Faraşin, Siirt Central, and Siirt Pervari regions. It reached almost the maximum

importance score (98.56%–100%) in all three regions, with an overall importance average of 99.52%. The second most important variable was His, which stood out with values of 76.03%, especially in Siirt Central and Siirt Pervari, and its overall average was 67.84%. The variables Arg (57.38%), Glu (53.89%), and Gly (48.43%) also contributed significantly to the model's discrimination power.

Table S6. Importance Levels of Phenolic Compounds in the SVM

Variable (Phenolic Compound)	Şırnak Faraşin	Siirt Merkez	Siirt Pervari	Overall
p-CA	100.00	69.82	100.00	89.94
Hesperetin	80.25	80.25	69.14	76.54
Luteolin	45.68	76.47	76.47	66.21
DBA34	62.96	58.30	62.96	61.41
DB34	26.54	49.07	49.07	41.56
Gentisic acid	38.55	38.55	31.48	36.19
Chrysin	41.36	20.51	41.36	34.41
HGA	31.48	29.84	31.48	30.93

Genistein	32.51	32.51	22.26	29.09
t-CA	32.10	20.51	32.10	28.24
Vanillic acid	15.50	26.34	26.34	22.73
Rutin	11.11	22.26	22.26	18.54
Pyrogallol	6.79	15.84	15.84	12.83
Quercetin	14.40	14.40	3.02	10.61
Vanillin	2.88	8.26	8.26	6.47
Caffeic acid	3.43	3.43	0.00	2.29

When the importance levels of phenolic acid compounds in the classification were examined as a result of Support Vector Machines (SVM) analysis, p-CA stood out as the compound with the highest average importance (89.94%) in terms of all regions. p-CA had the highest value with 100% in the Şırnak Faraşin and Siirt Pervari groups, while it was determined as 69.82% in Siirt City Center. Hesperetin (76.54% overall importance) and luteolin (66.21%) were identified as other high importance compounds. When evaluated on a regional basis, p-CA (100%), hesperetin (80.25%), and DBA34 (62.96%) stood out in the Şırnak Faraşin samples, while hesperetin (80.25%), luteolin

(76.47%), and p-CA (69.82%) stood out in Siirt City Center. In Siirt Pervari samples, p-CA (100%), luteolin (76.47%) and DBA34 (62.96%) were the most prominent compounds.
